# The 3Rs of Cell Therapy

**DOI:** 10.5966/sctm.2016-0180

**Published:** 2016-08-08

**Authors:** Arnold I. Caplan, Chris Mason, Brock Reeve

**Affiliations:** ^1^ Skeletal Research Center, Department of Biology, Case Western Reserve University, Cleveland, Ohio, USA; ^2^ Advanced Centre for Biochemical Engineering, University College London, London, United Kingdom; ^3^ AvroBio Inc., Cambridge, Massachusetts, USA; ^4^ Harvard Stem Cell Institute, Cambridge, Massachusetts, USA

## Abstract

The 3Rs for a good education are “reading, 'riting, and 'rithmetic.” The basis for good health care solutions for the emergent field of cell therapy in the future will also involve 3Rs: regulation, reimbursement, and realization of value. The business models in this new field of cell therapy will involve these 3Rs. This article brings forth realities facing this new industry for its approaches to provide curative health care solutions. Stem Cells Translational Medicine *2017;6:17–21*

**Significance Statement**

Cell therapies (including mesenchymal stem cell‐related technologies) are forecasted to substantially change disease outcomes and thus patient lives. The key to the speed of such adoption will be the relative success of companies to manage the new “3Rs”: regulation, reimbursement, and realization of value. This new industry will progress as technology develops and clinical experience accumulates, but only if the marketplace, regulatory and reimbursement agencies, and third‐party payers enable it by fashioning supportive, streamlined routings for getting these new therapies efficiently, and safely, to patients.

## Introduction

Cell therapy involves the introduction of live cells directly from the patient or from an exogenous source into tissues or the bloodstream to affect a therapeutic outcome. The cells may be used alone (cell therapy) or in combination with a scaffold (tissue engineering). The technology has been in play since the mid‐1950s, when hematopoietic bone marrow was first successfully transplanted to repopulate patients previously exposed to depopulating chemotherapy; the first recorded bone marrow implantation took place in Ulster, Ireland, in about 500 BC 1. Further back in time, rudimentary cell therapies have been used for thousands of years if one considers aspects of animal husbandry 2. In the 21st century, embryonic and adult cells, both fresh and culture‐expanded, allogeneic and autologous, have been used in various medical circumstances 3. The science had progressed sufficiently so that by the 1980s and 1990s many companies had started to produce tissue‐engineered skin substitutes or mesenchymal stem cells (MSCs) for clinical conditions 4, 5. Now, in the 2010s, companies are in the clinic with additional mature and progenitor cell types (such as neural cells, retinal cells, cardiac cells, and pancreatic cells) from a variety of sources for a broad set of disease states. Not considered here are the businesses that deal with hematological diseases, the manipulation of hematopoietic cells or their descendants, or gene therapy.

Whether the cells in question are from an autologous or allogeneic source, cell therapy as a clinical solution presents a business model challenge, especially in an environment that is dominated by large, highly successful pharmaceutical corporations that are used to selling “blockbusters”: high‐volume, low‐cost goods and high‐margin off‐the‐shelf products. Cell therapies are far from being in the “blockbuster” space, being low‐volume and costly to manufacture, whether they are individually made autologous therapies (more akin to a service) or universal allogeneic products. Now that the science and translation of cell therapy have advanced, the business model questions have extended beyond the allogeneic versus autologous debates of the last few years to a broader set of issues that get to how such products are approved, how the health authorities see their economic value relative to that of other solutions, and how companies will deliver on that value.

The historic precedents for value propositioning have been set in the biotech era of the 1980s with the emergence of Genentech, Amgen, and Biogen, to name but a few. These new corporations followed the business pattern established previously for small‐molecule drugs that produced major health care gains. The production of vaccines in the 1940s and 1950s had a profound effect in setting the business tone for the biotech companies of the 1980s. Indeed, the failure to produce safe polio vaccines 5, 6 was one of the primary drivers for the formation of the U.S. Food and Drug Administration (FDA) and its current central impact on economics and business strategies for new products for medical care delivery.

## The 3Rs

In the past century, it was widely said that the basis of a good education was the 3Rs of “reading, 'riting, and 'rithmetic.” In today's world of high health care costs (≥17% of U.S. gross domestic product) and a plethora of new, exciting technologies, the basis for good health care solutions can also be thought of as the 3Rs: regulation, reimbursement, and realization of value. Both public sector (especially the National Institutes of Health) and private funding have led to the invention and development of several medically driven sectors. Products in the device sector, such as implantable devices in orthopedics and cardiology, have led to an increase in longevity and have solved clinical problems of substantial scope and proportions. Corporations making such products have been allowed to fast‐track their products, often without the need for a clinical trial through the device‐specific 510(k) route. Most new medical devices enter the market via this route, which requires only demonstration of “substantial equivalence” to a previously marketed device. For example, in this context, more than 1 million knees and hips will be replaced with metal devices in 2014, and that number is predicted to increase by 10%–20% per year with the entrance of the baby boomers into the age range needing joint replacement. However, with the emergence of cell therapy potentially enabling joint tissue regeneration, this device segment may shrink during the coming years. Given the potential of cell therapy solutions to have long‐lasting, even curative, effects and given the inherent complexity of manufacturing and delivering such solutions to patients, paying close attention to the 3Rs will be even more important for companies trying to bring cell therapies into the health care marketplace than it is for the other three therapeutic pillars of health care: small‐molecule drugs, biologics, and medical devices 7.

## Exemplar: MSCs

The cell therapy industry is facing many unique challenges. MSCs will be used as an exemplar for the sector as a whole to illustrate the requirement for novel business models, as well as regulatory and reimbursement challenges, to enable these potentially game‐changing therapies to deliver transformative or curative therapies as part of everyday clinical practice.

MSCs reside in every tissue of the body as perivascular cells (pericytes) and function naturally at sites of blood vessel breakage or inflammation 8, 9, 10. From the front of the newly released and activated MSCs, a curtain 11 of biofactors is secreted that inhibits the overaggressive immune system from surveying the damaged tissue (the first line of defense against the establishment of autoimmune reactions). From the back of the MSC, trophic factors 12 are secreted that inhibit ischemia‐caused apoptosis, inhibit scar formation, stimulate angiogenesis, and stimulate the mitosis of tissue‐specific progenitors. The molecular mechanisms for these activities and functions are becoming known 13.

More than 600 clinical trials using MSCs (as shown on http://www.clinicaltrials.gov with “mesenchymal stem cells” used as the search term) are in progress around the world for clinical conditions such as multiple sclerosis, amyotrophic lateral sclerosis, stroke, acute and chronic heart failure, rheumatoid arthritis and osteoarthritis, kidney or liver fibrosis, spinal cord cuts or contusions, and sepsis. Thirty to forty corporations using various formulations of MSCs or MSC‐like cells from multiple tissue sources for various clinical indications have emerged. One of the biggest companies, Mesoblast Ltd., has a market capitalization of more than $1 billion and has recently purchased the cell‐therapy products and intellectual property from Osiris Therapeutics, Inc. (the first MSC company, founded in 1992). But in the face of challenging approval pathways and in the wake of unexpected adverse reimbursement changes, such as those encountered by Organogenesis and Dendreon in 2015, a key question remains: What is the pathway to success for the MSC products and the companies that are bringing them and similar cell therapy technologies forward?

## Regulation

Like any small‐molecule drug or biologic, a cell therapy must satisfactorily demonstrate safety and positive therapeutic effects in preclinical animal models, after which it transitions into human testing as a component or product to be tested in clinical trials under the auspices of a for‐profit company or, in academia, in an investigator‐initiated clinical trial. Indeed, the first‐in‐humans MSC therapy was conducted at Case Western Reserve University and University Hospitals of Cleveland in an investigator‐initiated study 14.

In either case, the standard pathway for the testing and acceptance of any new therapy in humans has been established by the sequential stepwise process of phase I, II, and III clinical trials. This process has its roots in the days of big pharma before the entry of biologics; the process then adapted to accommodate the biologics. These same procedures and outcome measures that were established for small‐ and macro‐molecule drugs are now used by national regulatory agencies to assess and approve cell therapies. But unlike drugs, whose structure, potency, and purity can be routinely documented, cell therapies are not so easily characterized because cells are complex multicomponent entities. This means that no standard regulatory route is now in place that is entirely appropriate, let alone favorable, for cell therapy.

The current guidelines for certification of cells for therapeutic use attempt to mimic aspects of the criteria long established for drugs and, consequently, bring with them several problems because they are not “fit for purpose.” The first problem is one of scope. The standard phased clinical trials have been set up by large, multibillion‐dollar pharmaceutical companies that have the resources to conduct such trials, some of which can cost hundreds of million dollars all‐in. Small companies specializing in cell therapy do not have that wherewithal. As a consequence, many clinical studies to date have been uncontrolled and underpowered, leading to anecdotal results, unclear benefits, and, often, failure in subsequent phases with larger patient populations.

Second, one can analyze and characterize a chemical or biologic drug to prove its composition, purity, and consistency of manufacturing lot. Defining and certifying the purity and composition of a group of living cells and ensuring that consistency over time is not so easy and, in many cases, is not 100% possible. Furthermore, in many cells, let alone mixtures of cell populations, one may not know exactly which components of the cell are critical and efficacious for a specific clinical indication.

Third, unlike a drug that is metabolized and excreted, cells may continue to live on in the body. Therefore, the regulatory authorities are right to be concerned about understanding what the cells do and where they go in the body (i.e., issues of homing, engraftment, cell division, and tumorigenicity that are nonissues for conventional drug products). For some cell types, such as MSCs, that may not live long in the body and for which there is sufficient clinical history of safety in the clinic, this will be less of an issue than for others. For many cell preparations, however, clinical approval may be dependent on other technologies, such as sophisticated in vivo tracking, which can be problematic especially for a small, resource‐constrained company.

Not addressed here are the questions of how to “tune” therapeutic cells, such as MSCs, to be optimal for the disease being treated and optimal for each patient. Currently, companies tend to use one batch of MSCs for all clinical situations and thus can be expected to have high “nonresponder” rates because of the lack of disease‐specific tuning.

In short, the current regulatory process can appear long, expensive, and disproportionately regulated, especially given that several cell therapies appear to be transformative and in some cases curative, but the FDA has been receptive to criteria proposed by different companies and organizations with new proposals for judging the efficacy and therapeutic potential of cell therapies. One such new process, recently instituted in Japan under their new Regenerative Medicine Act, enables a rapid (2‐ to 3‐year) route to conditional time‐limited approval with reimbursement. This requires an initial study to demonstrate clear safety and, at a minimum, a suggestion of efficacy 15. Full approval is subject to ongoing monitoring and longer‐term studies. Such innovative regulation is essential for the field to flourish. The first product has just emerged successfully through this route: HeartSheet (autologous skeletal myoblast sheets) from Terumu (Tokyo, Japan, http://www.terumo.com), with a reimbursement price of approximately $120,000. This and other types of new processes must be tailored to not only the new emerging technologies but also the limited resources of small corporations or academia because that is where most new cell‐based therapies are being developed and first tested in humans.

In the U.S., one provision of the Regenerative Medicine Promotion Act, introduced in March 2014, was to direct the Department of Health and Human Services to establish a Regenerative Medicine Coordinating Council, with one of its goals being development of “consensus standards regarding scientific issues critical to regulatory approval of regenerative medicine products.” In the meantime, in early November 2014, the FDA released new draft guidelines for human cells, tissues, and cellular and tissue‐based products to clarify what constitutes “minimal manipulation” for a cell therapy. Minimal manipulation of a cell population has been a key criterion for determining whether a given cell therapy is deployed under the practice of medicine or has to undergo the lengthier and more complex route of a traditional biologics license application. Clarity of definition and consistency around the world will be useful for the field because there is considerable confusion among all the stakeholders. However, in February 2015 the FDA started to progress the debate by issuing draft guidelines 16.

Last, because regulatory bodies change relatively slowly in response to the introduction of new therapies, it may be useful for legislative bodies to take the lead in effecting regulatory change. Certainly, the legislation brought forth in Japan has all the world watching its progression into product approvals for cell and gene therapies. Groups such as the Bipartisan Policy Center in Washington are exploring ways to have the U.S. Congress pass progressive legislation for cell‐based therapy (http://www.bipartisanpolicy.org; a conference titled “Advancing a New Policy Framework for Regenerative Cell Therapy” was held in April 2016). If successful, new legislation will enhance the FDA's regulatory capacity by settling both regulatory and societal goals. A proposal in this regard has been made previously 17.

## Reimbursement

To state the obvious, for a company to produce a health care product on an ongoing basis, it must be paid for and the company must be able to make a profit. Although in theory the health care system in the U.S. gives great leeway to producers to set price and determine value of a given therapeutic, in practice it puts huge control capacity in the hands of insurance companies and government agencies (especially the Centers for Medicare & Medicaid Services) to set the monetary standards for specific procedures and therapies. This is even more stringent in countries, such as the United Kingdom, that have explicit cost‐effectiveness controls in place through bodies such as the National Institute for Health and Care Excellence, where comparator based cost‐effectiveness may be hard to prove for early stage therapies.

For the foreseeable future, cell therapies will continue to be high priced because the cost to produce the large numbers of cells needed for a given therapy is substantial and the production runs are relatively small, with high production costs. However, the cost‐of‐goods can be expected to come down for several major reasons. First, future generations of bioprocessing tools, disposables and reagents, and acquired experience will reduce the cost of manufacturing. Second, increasing cell potency and the development of improved targeting strategies will lower the number of cells needed for a specific therapy, further reducing cost and variation. Third, economies of scale will begin to have a major impact in much the same way as has occurred for other drug platform technologies in the past (e.g., penicillin). Overall, this means that in the current early stages of the cell therapy era, inefficiency must be paid for to ensure efficacy and proof of principle for some of these treatments. Once a collection of cell therapy is approved and put into practice, the marketplace will reward companies that can do “more for less” money. We can expect that new production and innovative cell‐delivery strategies will emerge exactly as new strategies did in the monoclonal antibody production business during the past 20 years.

Companies that provide therapeutic cells need to be paid for producing and making such therapies accessible. Large pharmaceutical companies may have the resources to wait to obtain compensation should marketing approval come long after initial regulatory approval, but small companies do not have the same luxury. Mechanisms must be found to provide payment or reimbursement early in the approval process, provided there are the right contingencies regarding safety and efficacy. To date, some cell therapies have been approved by regulatory agencies, but reimbursement is still lacking. Japan's new legislation, as mentioned earlier in this article, which became effective at the end of November 2014, is an attempt to solve this conundrum. Many companies can be expected to take advantage of that. To date, Athersys, Cytori Therapeutics Inc., and Mesoblast Ltd. and others have set up shop in Japan to do so. Likewise, other governments and national regulatory bodies are observing the impact of the changes in Japan. Mesoblast's graft‐versus‐host product, Prochymal (marketed as Temcell by JCR Pharmaceuticals Co., Hyogo, Japan, http://www.jcrpharm.co.jp) was priced at the end of 2015 by the Japanese regulatory agency at approximately $7,000 per bag of 72 million MSCs (about 16–24 bags are used for a complete therapeutic course).

Another issue that will come to the fore in the cell therapy field is that many cell therapy solutions have the promise of treating the underlying cause of a disease. This is unlike many conventional drug products that manage the disease and/or its symptoms. If a therapy can affect a cure or a transformative change (e.g., a long‐term halt in disease progression), how is the company compensated for that? Currently, we pay for drugs and devices on an interventional basis, the potentially “once‐and‐done” approach deployed by cell and gene therapies is therefore a new challenge for reimbursement compared with the pay for a pill‐a‐day‐for‐life pharmaceutical practice. The recent discussion in the U.S. about the pricing of Sovaldi (Gilead, Foster City, CA, http://www.gilead.com)—$1,000 a pill, $84,000 for a 3‐month regimen—brought this issue out as the debate raged as to whether the value of avoided liver transplants was the appropriate determinant. Likewise, in Europe the pricing of Glybera (uniQure, Amsterdam, The Netherlands, http://www.uniqure.com/), at $1.4 million per treatment regimen, generated controversy.

Now that human pancreatic progenitor cell therapy is starting clinical trials, we can envision the time, for example, when β islet cell transplants for patients with diabetes removes the need for a lifetime of blood tests and insulin injections, not to mention avoiding the complications of the disease and their attendant costs. The latter are often twice the direct costs of the disease itself. In such a case, how does a company get reimbursed appropriately? Should it be based on the cost of the therapeutic itself or on the entire stream of value it creates, or somewhere in between? Recognizing that this value is created and captured only over time has led to proposed reimbursement plans whereby a company receives payment initially for the therapeutic intervention and on a periodic basis as the therapy proves out for an individual over time 18. This type of outcomes‐based compensation is attractive in that it aligns economic and health interests but will be difficult to implement in practice because it involves assignment of cause and effect and requires complex patient tracking and reporting over time, a special challenge in environments, such as the U.S., without a single‐payer system.

Again, for emphasis, the cost for these new therapies will be initially high, but once the product is established in the medical community, market pressure is sure to drive the price down. However, it is a mistake (not to be repeated) to assume that as more practitioners use an established cell‐based therapy, the costs per unit will decrease correspondingly. This was not the case for skin substitutes (Organogenesis Inc. did observe some economy of scale, but not enough) and is certainly not the case for most, if not all, cell therapies. The market will reward innovations that allow for more efficient cell production, tailoring cells for specific clinical situations so lower (optimal) doses can be used and patient‐specific assays (companion diagnostics) to ensure that proposed treatments are administered only to patients who are likely to benefit: a convergence of cell therapy and stratified medicine. In the drug industry, it is well known that not all patients will respond to a specific drug; in some cases, upwards of 30%–40% of the patients do not respond. In the case of hyaluronan injections into osteoarthritic knees, the nonresponder rate can be as high as 60%–70% of patients. These new approaches of successful cell therapies must have a rapid access to the market, together with reimbursement, much like the newly introduced Japanese conditional time‐limited approval. In these cases, long‐term follow‐up of (reimbursement) paid‐for products must be instituted.

## Realization of Value

All companies, large and small, have investors who expect a return on investment for both public and privately held companies. In the current marketplace, valuation of a company delivering therapeutics is an evolving process. One would think that as companies progress from one positive outcome to another at successive trial stages (i.e., reach significant inflection points), valuation would jump up, as is generally observed with small‐ and macro‐molecule drugs. However, this may or may not be true depending on the market conditions and the financial independence of each company. This is further complicated by the fact that there are insufficient data as of yet to determine whether cell therapies will have the same or different success rates at the various stages of development that drugs do (for which we have decades of data). For the time being, because new technologies are so difficult to price and to project actual market size and degree of penetration, reimbursement rates and regulatory paths are in flux, and we do not have a history of success rates in the development path, valuations tend to underestimate true worth.

In the cell therapy industry, the most dramatic indicators of value will be mergers and acquisitions, driven by both consolidation and companies looking to bring on new product lines. A strong driver for consolidation is the degree of overlap of various corporate intellectual properties and the strength and weakness of the two given companies’ balance sheets. This former consideration was certainly a strong component of why Mesoblast bought the cell therapy portfolio from Osiris Therapeutics Inc. Now Mesoblast has two overlapping but independently developed MSC products: their mesenchymal precursor cells and Osiris's MSCs. In all likelihood, these two products will be separately pursued by Mesoblast because of the regulatory approval process. As cell therapies prove their utility, we can expect more deals such as the Novartis investment in Gamida Cell, in which “big pharma” saw an opportunity to enhance its product line, as well as grow the market for umbilical cord blood transplants by making them more effective. Also, as more clinical data become available and the field learns which cells work best for which indications, we can expect to see more direct competition as companies compare their cell‐based solutions on a head‐to‐head basis as happens today with drug products. That will bring subsequent paring of product lines or ceding of selected market spaces as the companies “rationalize” on the basis of technology or market position.

## Conclusion

Cell therapies (including MSC‐related technologies) are forecasted to substantially change disease outcomes and thus patients’ lives. The key to the speed of such adoption will be the relative success of companies to manage the new 3Rs (regulation, reimbursement, and realization of value). This new industry will progress as technology develops and clinical experience accumulates, but only if the market place, regulatory and reimbursement agencies, and third‐party payers enable it by fashioning supportive, streamlined routings for getting these new therapies efficiently, and safely, to patients. The field should not take shortcuts, but it should be open to questioning long‐held assumptions and look creatively for new economic and regulatory mechanisms that can accelerate bringing these potentially transformative therapies safely to patients.

## Acknowledgments

We thank the L. David and E. Virginia Baldwin Fund for their financial support.

## Author Contributions

A.I.C., C.M., and B.R.: manuscript writing, final approval of the manuscript.

## Disclosures of Potential Conflicts of Interest

A.I.C. has received Case Western Reserve University royalties from Osiris/Mesoblast. C.M. is chief scientific officer, AvroBio Inc. B.R. is a Bipartisan Policy Center advisory working group member.
